# The impact of estrogen status on the gut microbiome: a systematic review and meta-analysis

**DOI:** 10.3389/fendo.2026.1780806

**Published:** 2026-04-02

**Authors:** Kristina Saravinovska, Daniele Santi, Francesco Costantino, Alessandro Prete, Antoan Stefan Šojat, Giorgia Spaggiari, Miomira Ivović, Irene Lambrinoudaki, Eleni Armeni, Aleksandar Jurišić, Sladjana Mihajlović, Svetlana Vujović, Ljiljana V. Marina

**Affiliations:** 1 Faculty of Medicine, University of Belgrade, Belgrade, Serbia; 2 Clinic of Endocrinology, Diabetes and Metabolic Diseases, University Clinical Centre of Serbia, Belgrade, Serbia; 3 Unit of Endocrinology, Department of Biomedical, Metabolic and Neural Sciences, University of Modena and Reggio Emilia, Modena, Italy; 4 Unit of Endocrinology, Department of Medical Specialties, Azienda Ospedaliero-Universitaria of Modena, Modena, Italy; 5 Unit of Andrology and Sexual Medicine of the Unit of Endocrinology, Department of Medical Specialties, Azienda Ospedaliero-Universitaria of Modena, Modena, Italy; 6 Department of Metabolism and Systems Science, School of Medical Sciences, College of Medicine and Health, University of Birmingham, Birmingham, United Kingdom; 7 Centre for Endocrinology, Diabetes and Metabolism, Birmingham Health Partners, Birmingham, United Kingdom; 8 NIHR Birmingham Biomedical Research Centre, University of Birmingham and University Hospitals Birmingham NHS Foundation Trust, Birmingham, United Kingdom; 9 2nd Department of Obstetrics and Gynaecology, University of Athens, Aretaieio Hospital, Athens, Greece; 10 Department of Diabetes and Endocrinology, Royal Free Hospital NHS Foundation Trust, London, United Kingdom; 11 Clinic for Gynecology and Obstetrics “Narodni Front”, Belgrade, Serbia; 12 Department of Obstetrics and Gynecology, University Hospital “Dragisa Misovic”, Belgrade, Serbia

**Keywords:** estrobolome, gut microbiome, gut microbiota, hypoestrogenic, menopause, postmenopause, premature ovarian insufficiency

## Abstract

**Background:**

Estrogens have been proposed as modulators of gut microbiome (GM) composition, yet evidence from observational studies remains inconsistent.

**Objective:**

This meta-analysis aimed to systematically summarise existing evidence on GM alterations in hypoestrogenic women – post-menopausal or premature ovarian insufficiency (POI) – compared to euestrogenic pre-menopausal controls.

**Methods:**

PubMed, SCOPUS and Embase were searched through December 2024 for studies comparing GM characteristics between hypoestrogenic and pre-menopausal women. Primary outcome was α-diversity (Shannon index). Secondary outcomes included relative abundances of *Bacteroidetes*, *Firmicutes*, and the *Bacteroidetes* to *Firmicutes* ratio. Random-effects models were used for data synthesis.

**Results:**

Out of 1092 studies screened, 7 met the inclusion criteria (n = 45 women with POI, n = 1222 post-menopausal women, n = 463 eustrogenic controls). No significant differences were observed in *α*–diversity (p=0.990), *Bacteroidetes* (p=0.440), or *Firmicutes* abundance (p=0.110) between hypoestrogenic and euestrogenic groups, irrespective of POI or postmenopause. Similarly, the *Bacteroidetes* to *Firmicutes* ratio showed no significant difference between the groups (p=0.400). Study heterogeneity was high (I² 61-99%).

**Conclusion:**

Current evidence does not support consistent differences in GM diversity or major bacterial phyla between hypoestrogenic and euestrogenic women. Given the substantial heterogeneity, limited control of confounding factors, and variability in methodological quality, these findings should be interpreted with caution. High-quality, well-controlled studies are needed to better define the relationship between estrogen status and the GM.

## 1 Introduction

Interest in the gut microbiome (GM) and its potential influence on health has expanded rapidly in recent years (1–4). The GM is a complex and dynamic ecosystem shaped by host biology and environmental factors, including age, diet, lifestyle, and hormonal status (5–10). In women, reproductive ageing represents a major transition, characterized by profound changes in circulating estrogen levels. Because estrogens have immunomodulatory, metabolic, and epithelial effects, it has been hypothesised that shifts in estrogen status – particularly during menopause or premature ovarian insufficiency (POI) – may be reflected in alterations in GM composition.

**Figure 1:**
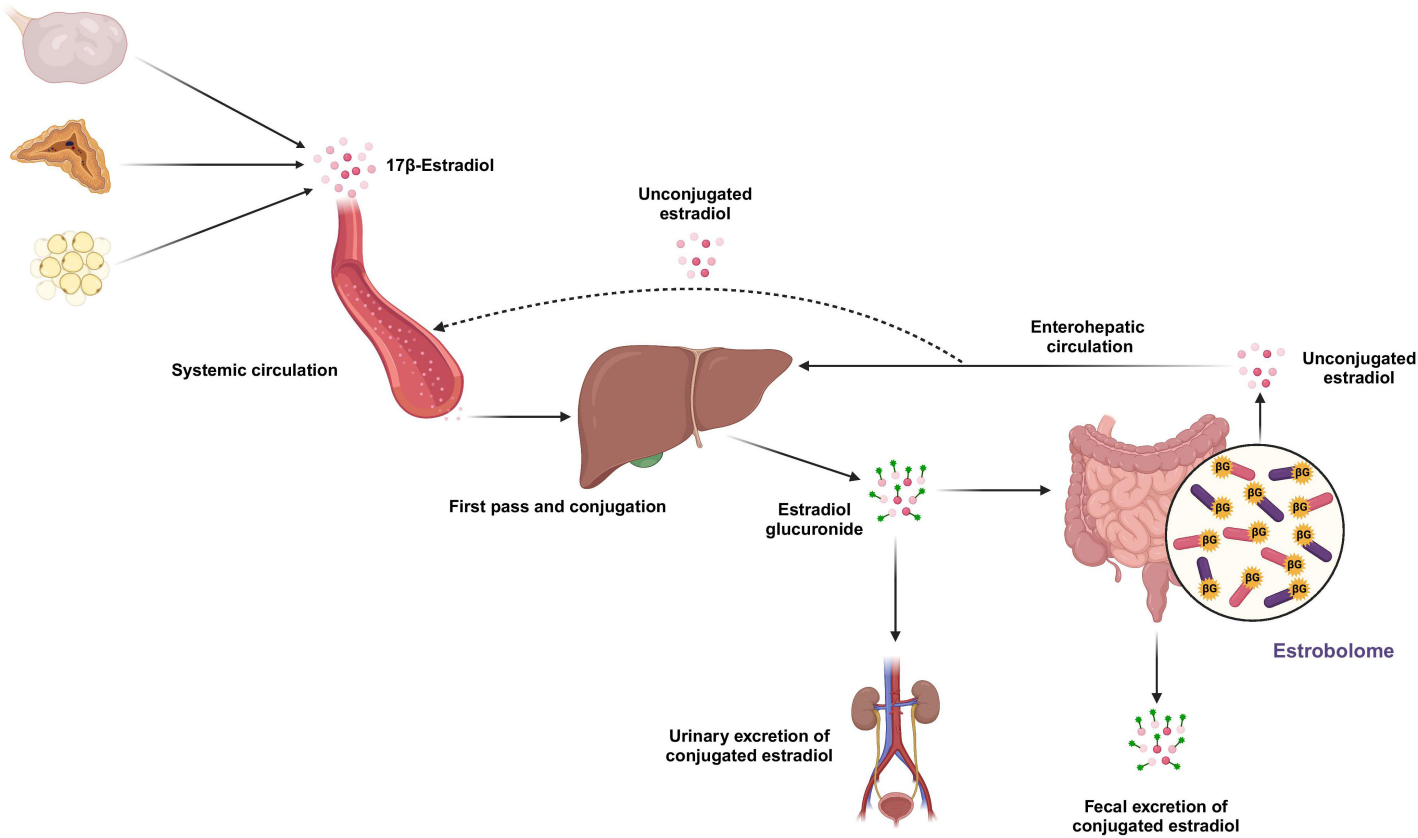
Overview of the Estrobolome. Endogenous estrogens are mainly secreted by the ovaries, adrenal glands, and adipose tissue. After entering systemic circulation, they reach the liver where they undergo first-pass metabolism and conjugation, forming glucuronidated estrogens. A portion is excreted in urine, while another portion enters the intestines, where some are deconjugated by β-glucuronidase-producing bacteria, resulting in unconjugated estrogens. These are reabsorbed into systemic circulation via enterohepatic circulation, thus increasing circulating estrogen levels. *Created in BioRender. Sarav, K. (2024)* *https://BioRender.com/y23y384*.

Mechanistically, the relationship between estrogens and the GM has been attributed in part to the “estrobolome”, a collection of bacteria-driven enzymatic reactions involved in gut estrogen metabolism (11–14) (**Figure 1**). This process facilitates entero-hepatic recirculation of estrogens and has been proposed as a potential mechanism by which the GM may modulate estrogen availability. Conversely, estrogens may influence GM composition by influencing gut barrier integrity, immune response, and microbial niche conditions (15–17). These bidirectional interactions have prompted hypoestrogenic states may be associated with dysbiosis.

Despite this growing interest, studies directly comparing the GM of hypoestrogenic women (post-menopausal or POI) with euestrogenic pre-menopausal controls have produced inconsistent results. Some small-scale studies have reported reduced α-diversity (which includes the Shannon Index, reflecting species diversity) among post-menopausal women when compared to menstruating women, while others have demonstrated minimal differences (18–20). In POI, findings have also varied, with some reports suggesting specific taxonomic perturbations or decreased β-diversity (17, 21). Importantly, many of these investigations are limited by small sample sizes, heterogeneous populations, inconsistent exclusion of known GM disruptors (e.g., obesity, diabetes, antibiotics, probiotics, smoking), and variability in sequencing methods and analytic pipelines. The most commonly used parameter to assess differences between bacteria groups is α-diversity, which includes the Shannon Index as a metric and reflects species diversity within a specific ecosystem, combining species richness and evenness. Whereas β-diversity measures the change in species diversity between ecosystems and can be used for ecosystem comparison (22).

In the current literature, no systematic revisions with meta-analysis on GM in women with hypoestrogenism involving postmenopausal and women with POI are available. In this study, we aimed to systematically review and combine existing data to understand the alterations in GM changes in both postmenopausal and women with POI.

## 2 Materials and methods

The study was conducted according to a predesigned protocol, developed in conformance with the 2015 PRISMA (Preferred Reporting Items for Systematic reviews and MetaAnalyses) statement (**Supplementary File 1**) [52]. The meta-analysis was *a priori* registered in the international prospective register of systematic reviews (PROSPERO) database (ID CRD42024497630).

### 2.1 Search strategy and study selection

We did a comprehensive electronic search of the PubMed, SCOPUS and Embase library databases forstudies published from inception until December 23, 2024 using the following terms:(“premature ovarian insufficiency” OR “POI” OR “menopause” OR “post-menopause”) AND (“gut microbiome” OR “gut microbiota” OR estrobolome) (**Supplementary File 2**). Postmenopause and POI were defined as loss of ovarian function in women above or under 40 years of age, respectively. We included full-English-text, original, observational studies regarding GM composition in hypoestrogenic compared to euestrogenic women. We further searched bibliographies of included articles to identify any eligible studies that the electronic search may have missed. Studies reporting on hormonal replacement therapy (HRT) use or women with active infections, active intestinal diseases, or history of cancer were excluded.

Two reviewers (KS and FC) independently screened identified studies for eligibility. Conforming to the predefined inclusion criteria, they reviewed the titles and abstracts of identified studies in duplicate and removed all studies that did not fulfill the inclusion criteria at this stage. When reviewers disagreed, studies progressed to the next stage. In this phase, the same reviewers independently screened full-text articles to assess eligibility for final inclusion. When there was any conflict, it was solved by two co-agreeing investigators (LM and DS).

### 2.2 Data extraction and quality assessment

Data extracted from each study included first author, year of publication, country where the study was conducted, recruitment period, definition of hypoestrogenic status, inclusion/exclusion criteria, population recruited (including age at recruitment, age at menarche and menopause, years since menopause, body mass index [BMI], waist-hip circumference [WHR], smoking and alcohol drinking habits, number of previous pregnancies), potential confounders, GM profiling method, and relevant results (including GM characteristics, pituitary-gonadal axis hormone levels). Data extraction was performed by two reviewers, KS and FC. Studies were divided between the reviewers, with each reviewer independently extracting data from their assigned studies using a standardized data extraction form. Any uncertainties or ambiguous data were discussed between reviewers and resolved by consensus. When studies were considered eligible, but data were incomplete in the article or in the **Supplementary Materials**, corresponding authors were contacted *via* email to obtain missing data.

The primary endpoint was *α*-diversity, evaluated by the Shannon index, comparing hypoestrogenic women (study group) to euestrogenic pre-menopausal women (control group). The α-diversity index is considered as a closer proxy of intestinal dysbiosis and measures species heterogeneity in a single sample. When this index was not available, it was calculated at the species level using all the identified species reported in the **Supplementary Materials** with the formula H=−∑[(pi)×log_e_(pi)], where: H, Shannon diversity index; pi, proportion of individuals of one particular species in the whole microbiota community; ∑, sum (23). When studies reported the median and the interquartile range (IQR) of the index, the corresponding mean ± standard deviation (SD) was calculated [54, 55]; meanwhile, when articles reported the standard error of mean (σ), SD was calculated using the formula SEM = σ/√*n*; σ = SEM × √*n* (1), where *n* indicates the number of subjects. Secondary endpoints were β-diversity (species diversity between different samples), *Firmicutes, Bacteroidetes* and other phyla relative abundances, and *Bacteroidetes to Firmicutes* ratio.

Two reviewers (DS and FC) independently assessed the quality of each included study, using the Newcastle-Ottawa scale for observational studies. This scale relies on a 9-star system in which scores of 0–3, 4–6, and 7–9 are considered poor, moderate and good quality, respectively [56].

### 2.3 Data synthesis and statistical analysis

Heterogeneity among studies (I^2^) was considered as “low,” “moderate,” and “high” for values of 25, 50, and 75%, respectively [57]. Considering the high heterogeneity expected for the outcomes selected, the random effect model was applied to evaluate the mean difference (MD) among continuous data.

Subgroup analyses were performed considering if the hypoestrogenism was due to POI or to post-menopause, and based on the sequencing method, shotgun metagenomic sequencing or 16S rRNA gene sequencing. To address the potential confounding due to oral contraceptive use reported in premenopausal participants in one study (19), a subanalysis removing this study was conducted. If a significant difference was detected between post-menopausal/POI and pre-menopausal women, or between shotgun metagenomic sequencing or 16S rRNA gene sequencing, meta-regression analysis was performed, considering other endpoints extracted. Since meta-regression is typically recommended only when there are ≥10 studies per covariate, analyses including fewer studies were considered purely exploratory. The meta-regression analysis result was synthesized reporting both slope (S) and intercept (I) with appropriate lower and upper limits.

The Review Manager (RevMan) 5.3 software (Version 5.3.1 Copenhagen: The Nordic Cochrane Centre, The Cochrane Collaboration, 2014) was used to perform meta-analyses. Meta-regression analyses were performed using Comprehensive Meta-analysis Version 2, Biostat (Englewood, NJ, USA). Statistical significance was considered for *p* values < 0.05.

## 3 Results

**Figure 2:**
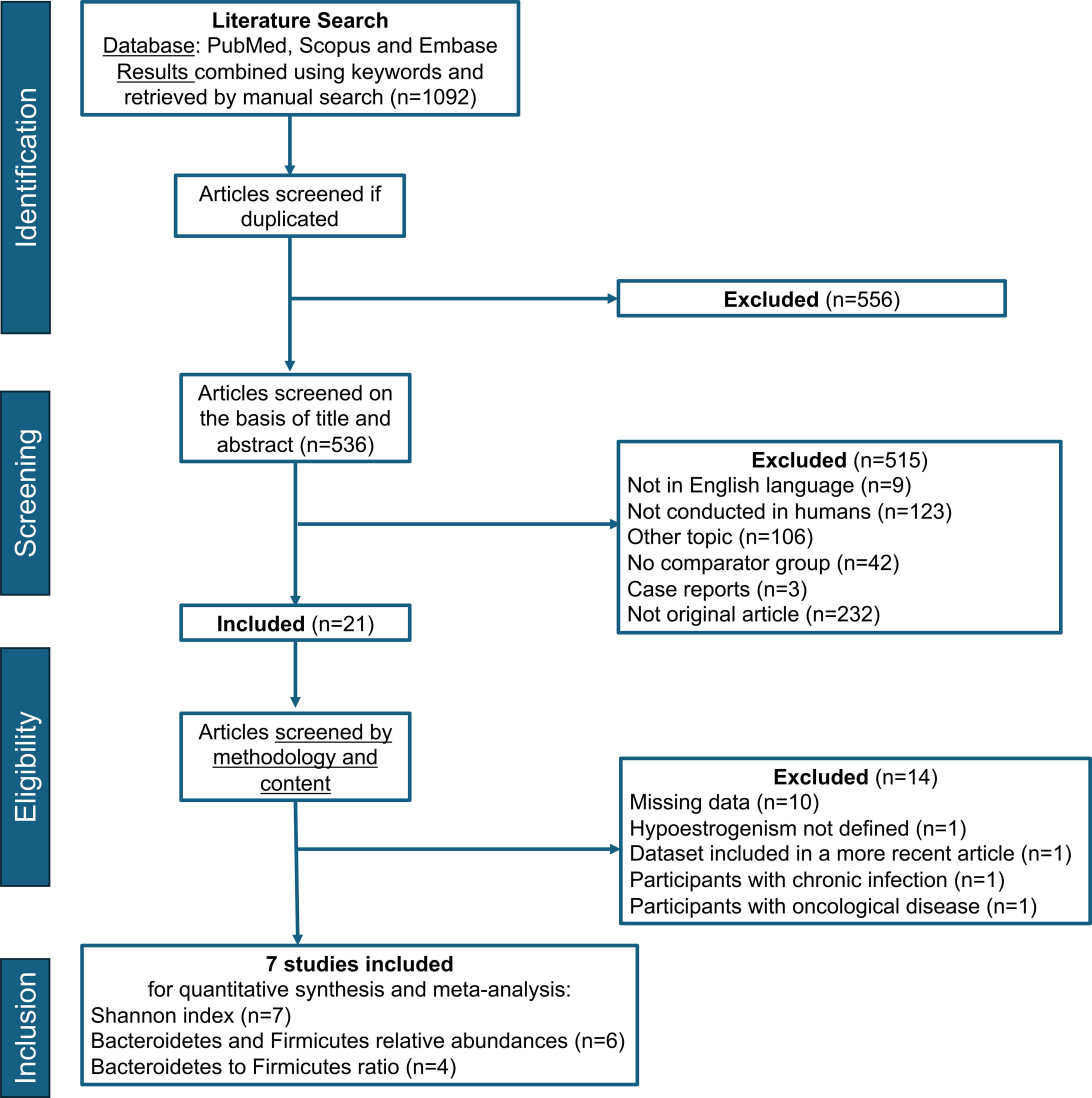
PRISMA flow diagram.

Among 1092 abstracts screened, 21 studies were assessed for eligibility (**Figure 2**). Fourteen studies were excluded, ten because the primary endpoint was not reported, one because the hypoestrogenism was not defined, one because the participants had chronic infection, one because the participants had an oncological history and one since it involved dataset published in a more recent article (**Figure 2**, **Supplementary File 3**). Seven studies were included in the meta-analysis (**Figure 2**). Four studies assessed the GM using shotgun metagenomic sequencing (18, 19, 24, 25), whereas three studies used 16s rRNA gene sequencing method (17, 21, 26). The characteristics of the included papers are summarized in **Table 1** (17–19,21, 24–26). The articles were published between 2018 and 2024 and had sample sizes in a range of 30 to 1322 patients. All studies were cross-sectional (17–19, 21, 24–26). Four were conducted in China (17, 18, 21, 24), two in the USA (19, 25), and one in Japan (26). The participants were pre-menopausal women (n = 463 in total) (17–19, 21, 24–26), post-menopausal women (n = 1222 in total) (18, 19, 24–26), and women with POI (n = 45 in total) (17, 21). Three studies were assessed as moderate risk of bias and four as low risk (**Supplementary File 4**).

**Table 1:** Study population characteristics.

Author (year)	Country	Period	Population included in the meta-analysis	Definition of hypoestrogenic states	Exclusion criteria	Confounders	Estrogen serum levels (pmol/L), Mean ± SD	Sampling and Sequencing		
								Sample	Sequencing method	Sequencing platform
Zhu et al. (2018) (24)	China	NA	25 pre-menopausal women 46 post-menopausal women	NA	Both groups: 1) Diarrhea, diabetes mellitus, ulcerative colitis, Crohn’s disease, or infectious diseases 2) Usage of antibiotics in the past 3 months 3) Usage of steroid hormones 4) Usage of probiotics or Chinese herbal medicine 3 months before	NA	NA	Feces	Shotgun metagenomic sequencing	Illumina HiSeq
Zhao et al. (2019) (18)	China	NA	24 pre-menopausal women 24 post-menopausal women	NA	Both groups: 1)History of chronic serious infection, any current infection and any type of malignant cancer 2) Usage of antibiotic treatment within 1 month before participating in the study Post-menopausal group: 1) other than natural menopause	Hypertension, Diabetes, Hyperlipidemia, Smoking, Alcohol intake, Diarrhea, Gastritis, Hyperthyreosis Fatty liver disease	NA	Feces	Shotgun metagenomic sequencing	BGISEQ-500 platform
Wu et al. (2021) (21)	China	From August 2019 to September 2019	18 pre-menopausal women 35 POI patients	POI: - primary or secondary amenorrhea for at least 4 months - at least two determinations of serum FSH > 40 IU/L with an interval of 4–6 weeks - before 40 years	Both groups: 1) Pregnancy 2) Tumor, chronic diarrhea, autoimmune diseases, gastrointestinal disease, active infections 3) Use of antibiotics/medications in the preceding 3 months 4) Chemo/radiotherapy 5) BMI< 18.5 or > 23.9 kg/m2 6) Smoking Pre-menopausal group: 1) normal ovarian function, 2) no history of menstrual dysfunction and infertility; 3)regular menstruation 4) FSH < 10 IU/L		Pre-menopausal group: 200.31 ± 33.04 POI group: 112.75 + 42.95	Feecs	16S rRNA (V3-V4)	Illumina NovaSeq
Jiang et al. (2021) (17)	China	NA	10 pre-menopausal women 10 POI patients 10 POI patients taking HRT	POI: - oligo/amenorrhea for at least 4 months, - FSH > 25 IU/L detected at two intervals more than four weeks apart - before 40 years old.	Both groups: 1) Infections, malignant tumors, intestinal diseases, obesity or other metabolism-related diseases 2) drug or alcohol use, and antibiotics, probiotics, or prebiotics use in the past three months	NA	Pre-menopausal group: 410.09 ± 72.62 POI group: 102.03 ± 24.86	Feecs	16S rRNA (V3-V4)	Illumina HiSeq
Yoshikata et al. (2022) (26)	Japan	From May 2021 to July 2021	35 pre-menopausal women 35 post-menopausal women	Post-Menopause: 1) No menstruation for 12 months; 2) FSH >25 mIU/mL; 3) E2 <20 pg/mL.	Both groups: 1) Having or being treated for genitourinary symptoms such as vaginitis and cystitis 2) those with unstable ovarian function	NA	Pre-menopausal women: 617.15 ± 968.44 Post-menopausal group: 36.7 + 0.00	Feces	16S rRNA (V1-V2)	NA
Peters et al. (2022) (19)	USA	From 2014 to 2017	295 premenopausal women 1027 postmenopausal women	Post.Menopause: 1) answered no to the question “Have your natural periods stopped permanently?”	Both groups: 1) Cancer history Postmenopausal group: 1) HRT or hormonal birth control 2) Other than natural menopause 3) <35 years old Premenopausal group: 1) > 55 years old 2) Did not have a period within 90 days prior to the visit 3) > 45 years old at the study visit with stool sample collected >2 years after the study visit 4) > 45 years at the study visit with stool sample collected <2 years after the study visit but did not have a period within 60 days prior to the visit	Obesity, Hypertension, Diabetes, Hyperlipidemia, Smoking, Alcohol intake, Antibiotics Oral contraceptive pills in the premenopausal group	NA	Feces	Shotgun metagenomics sequencing	Illumina NovaSeq
Wang et al. (2024) (25)	USA	From 2015 to 2019	56 pre-menopausal women 90 Post-menopausal women	Post-Menopause: 1) no periods for ≥12 months; not due to pregnancy or medication use 3) bilateral ovariectomy 4) uncertain status but age ≥55 years	Post-menopausal group: 1) taking hormonal contraceptives or HRT	Obesity, Hypertension, Diabetes, Hyperlipidemia, Smoking, Alcohol intake, Recreational drug use	NA	Feces	Shotgun metagenomics sequencing	Illumina NovaSeq

POI, Premature ovarian insufficiency; FSH, Follicle stimulating hormone; E2, Estradiol; NA, Not available in the original article.

**Figure 3:**
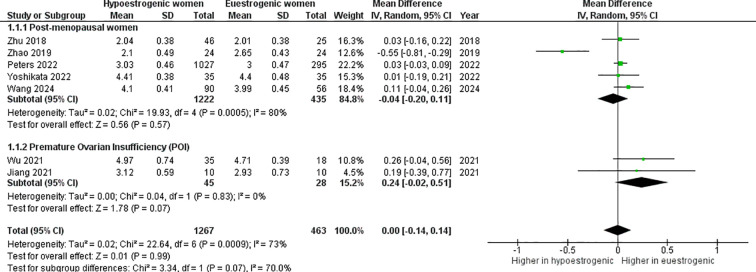
Forest plot showing the comparison of α–diversity index (Shannon index) between hypoestrogenic and euestrogenic women. SD, standard deviation; CI, confidence interval.

**Figure 4:**
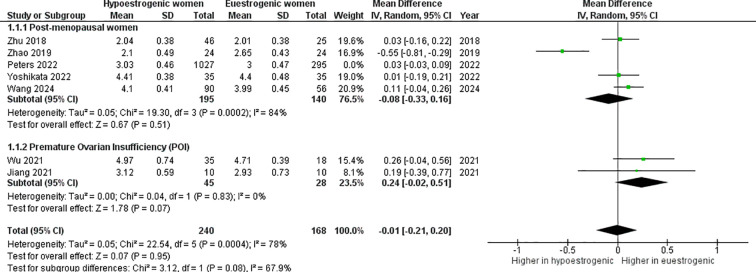
Forest plot showing the comparison of α–diversity index (Shannon index) between hypoestrogenic and euestrogenic women after removal of the Peters study. SD, standard deviation; CI, confidence interval.

**Figure 5:**
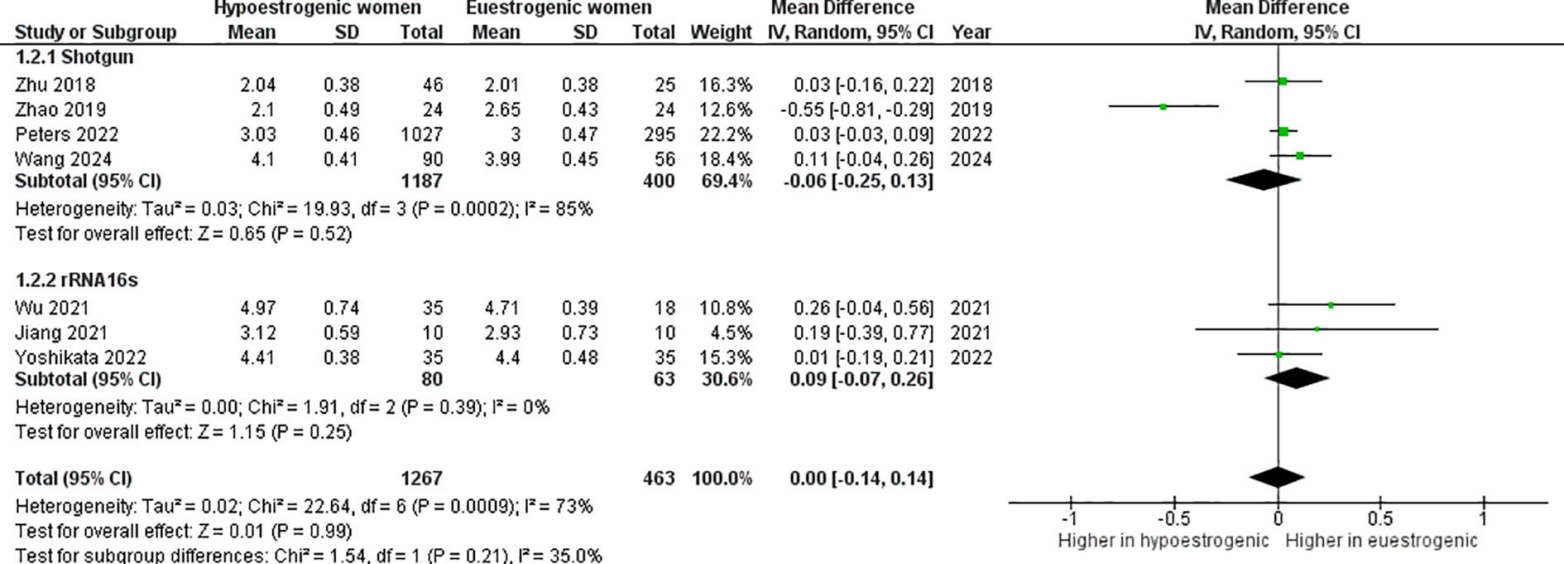
Forest plot showing the comparison of α–diversity index (Shannon index) between hypoestrogenic and euestrogenic women based on the sequencing method. SD, standard deviation; CI, confidence interval.

The *α*-diversity index was not significantly different between hypoestrogenic and euestrogenic women (p=0.990, I^2^ = 73%) (**Figure 3**). Subgroup analysis also showed a lack of statistical significance (POI *vs.* eustrogenic women, p=0.070, I^2^ = 0%; post-menopausal *vs.* eustrogenic women, p=0.570, I^2^ = 80%). This result remained also when the work by Peters et al. has been removed (mean difference -0.01; 95%CI: -0.21, 0.20, p=0.950) (**Figure 4**). Similarly, no consistent differences were found when dividing studies using shotgun metagenomic sequencing (mean difference -0.06; 95%CI: -0.25, 0.13, p=0.520) and 16S rRNA gene sequencing (mean difference 0.09; 95%CI: -0.07, 0.26, p=0.250) (**Figure 5**).

**Figure 6:**
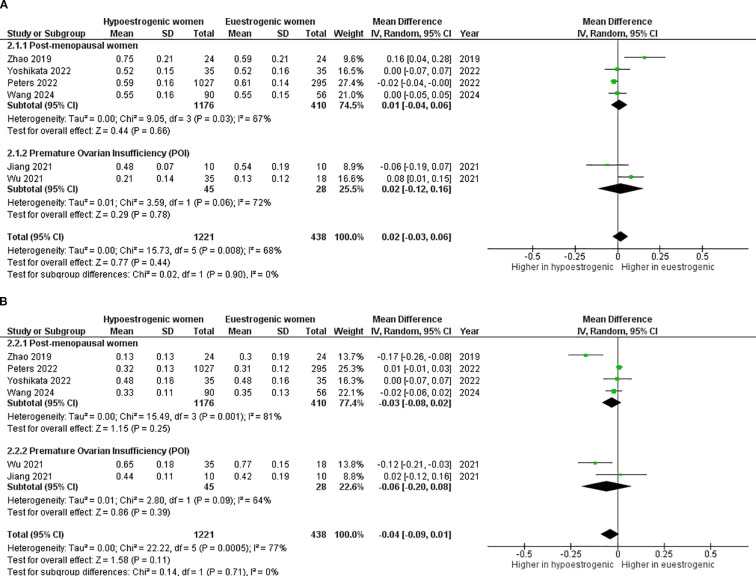
Forest plot showing the comparison of Bacteroidetes abundance **(A)** and Firmicutes abundance **(B)** between hypoestrogenic and euestrogenic women. SD, standard deviation; CI, confidence interval.

No significant differences were seen in *Bacteroidetes* (**Figure 6A**) and *Firmicutes* components (**Figure 6B**) between hypoestrogenic and eustrogenic women (p=0.440, I^2^ = 68% and p=0.110, I^2^ = 77%, respectively).

**Figure 7:**
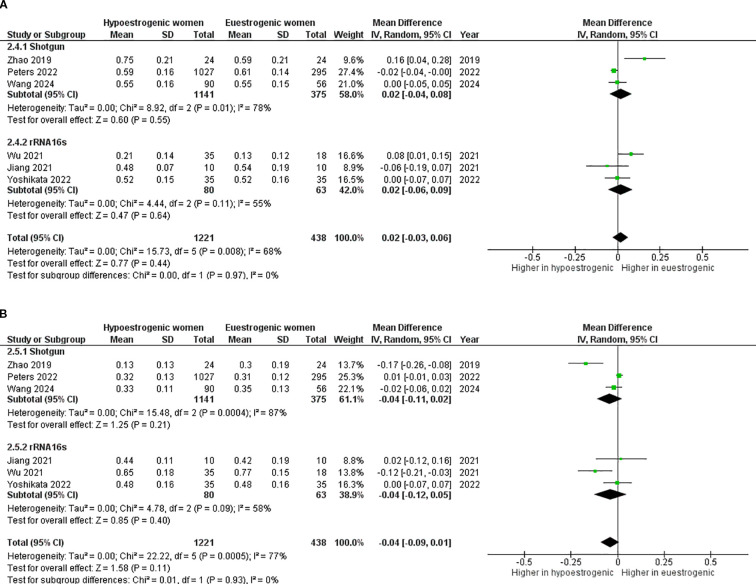
Forest plot showing the comparison of Bacteroidetes abundance **(A)** and Firmicutes abundance **(B)** between hypoestrogenic and euestrogenic women based on the sequencing method. SD, standard deviation; CI, confidence interval.

**Figure 8:**
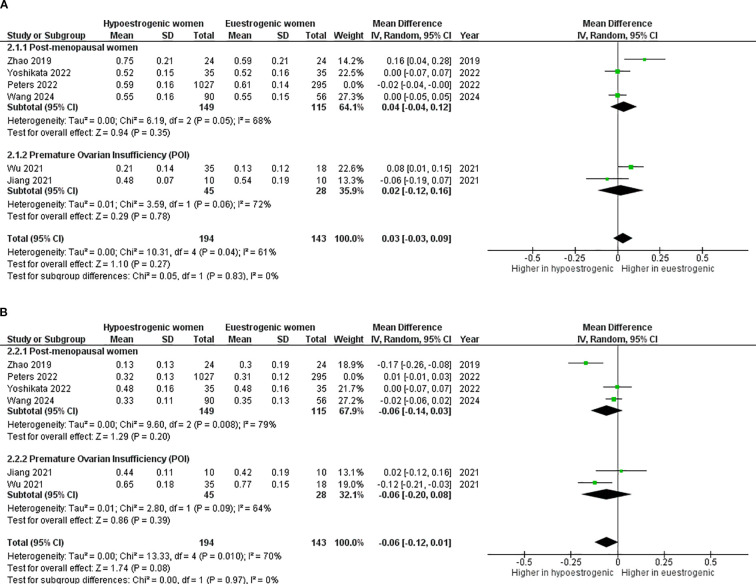
Forest plot showing the comparison of Bacteroidetes abundance **(A)** and Firmicutes abundance **(B)** between hypoestrogenic and euestrogenic women after removal of the Peters study. SD, standard deviation; CI, confidence interval.

Considering *Bacteroidetes*, the lack of difference remained also dividing studies using shotgun metagenomic sequencing (mean difference 0.02; 95%CI: -0.04, 0.08, p=0.550) and 16S rRNA gene sequencing (mean difference 0.02; 95%CI: -0.06, 0.09, p=0.640) (**Figure 7A**). Similar results were obtained for *Firmicutes* analysis (shotgun metagenomic sequencing - mean difference -0.04; 95%CI: -0.11, 0.02, p=0.210) and 16S rRNA gene sequencing (mean difference -0.04; 95%CI: -0.12, 0.05, p=0.400) (**Figure 7B**). This lack of significant difference was confirmed also in subgroup analyses, when Peters et al. was removed in both *Bacteroidetes* (mean difference 0.03; 95%CI: -0.03, 0.09, p=0.270) (**Figure 8A**) and *Firmicutes* analysis (mean difference -0.06; 95%CI: -0.12, 0.01, p=0.080) (**Figure 8B**).

**Figure 9:**
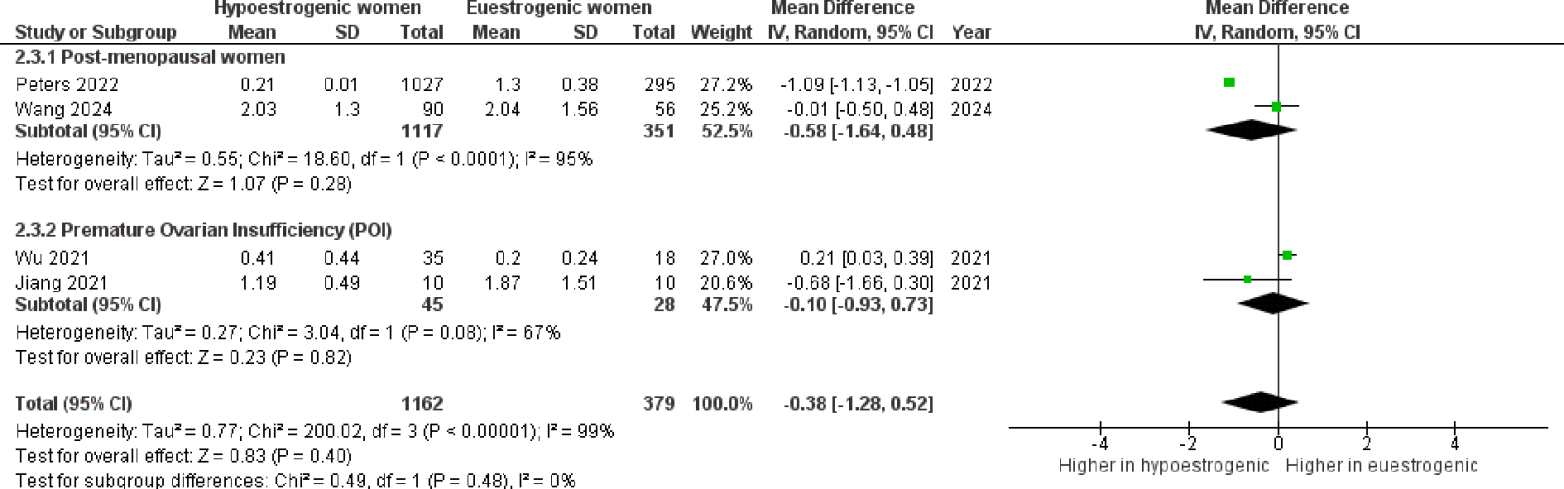
Forest plot showing the comparison of Bacteroidetes to Firmicutes ratio between hypoestrogenic and euestrogenic women. SD, standard deviation; CI, confidence interval.

Finally, considering the *Bacteroidetes* to *Firmicutes* ratio, no consistent differences were observed comparing hypoestrogenic to euestrogenic women (p=0.400, I^2^ = 99%), irrespective of POI and post-menopause (**Figure 9**).

## 4 Discussion

In this systematic review and meta-analysis, we found no significant differences in α-diversity, relative abundances of *Bacteroidetes* and *Firmicutes*, or the *Bacteroidetes* to *Firmicutes* ratio between hypoestrogenic and euestrogenic women. These results were consistent across subgroup analyses of post-menopausal women and women with POI, and across both shotgun metagenomic and 16S rRNA gene sequencing platforms.

The absence of clear differences contrasts with the hypothesis that declining estrogen levels contribute significantly to altered GM composition through the estrobolome. Although β-glucuronidase activity and enterohepatic recirculation of estrogens remain biologically plausible mechanisms of host–microbe interaction, the aggregated data suggest that these processes may not translate into reproducible, large-scale compositional changes detectable at the level of α-diversity or dominant phyla. Estrogen-related effects may be subtle, strain-specific, or functionally relevant without producing broad taxonomic shifts.

Similarly, while individual studies have reported diminished abundance of certain β-glucuronidase-expressing species (e.g., *Parabacteroides johnsonii, Clostridium lactatifermentans, Akkermansia muciniphila*) (19) and short-chain-fatty-acid-producing genera (*Roseburia*) in hypoestrogenic states (18, 19, 27), these findings have not been consistent across studies. Women with POI were found to have increased levels of *Eggerthella* in their feces, indicating a possible shift towards gut dysbiosis, which was reversed by stroprogestagen therapy (17). Supporting a possible causal role, mice administered *Eggerthella* showed signs of ovarian fibrosis and inflammation, which were ameliorated after introducing estradiol (17).

Differences in sequencing depth, taxonomic assignment, and statistical correction further complicate cross-study comparisons. Our results emphasise that proposed estrogen–microbiome interactions may not manifest through broad microbial diversity metrics but may instead involve narrower, functionally relevant pathways that require more refined analysis. Mouse models showed different caecal microbial flora based on sex hormone level, and estrogen receptor stimulation in male mice resulted in a significant reduction in the Shannon index and in the abundance of bacterial species known to influence insulin sensitivity (28), as well as a decline of *Proteobacteria* and a higher abundance of *Akkermansia* (29). All these findings suggest a bidirectional relationship between the steroid sex-hormone levels and the gut microbial ecosystem, where the diversity and abundance of GM depend on the level of sex hormones, while the activity of certain microbiome species modulates hormonal levels through deconjugation and entero-hepatic recycling.

The high degree of heterogeneity observed across analyses suggests that the lack of standardisation and variable population characteristics have likely obscured possible associations. Some of the included studies enrolled participants with conditions known to alter the GM – such as obesity (25), hypertension (18, 25), diabetes (18, 25), dyslipidemia (18), gastrointestinal disorders (18), alcohol consumption (18), and smoking (18) – yet did not consistently exclude or adjust for these factors. Given the established impact of these conditions on GM diversity and composition, they may represent stronger determinants of GM structure than estrogen status itself. Similarly, dietary intake and probiotic use, both major modulators of the GM, were insufficiently reported in most studies. Antibiotic exposure was inconsistently addressed (25, 26), and in some cohorts (24), the exclusion window may not have been adequate to fully mitigate its effects (30). These issues introduce substantial residual confounding and restrict the ability to attribute differences – or lack thereof – to estrogen status.

Geographical clustering also limits generalisability. Most studies were conducted in East Asia or North America, with relatively homogenous dietary patterns, genetic backgrounds, and lifestyle factors within cohorts. Given the profound effect of geography and diet on the GM, the evidence base lacks broader representation from other regions, limiting the generalisability of our findings (31, 32).

An important limitation of the evidence is the reliance on α-diversity and phylum-level measures. Although widely used, these metrics may not adequately capture microbial function or strain-level variation, nor do they necessarily reflect dysbiosis. A recent international consensus discouraging the use of the Bacteroidetes to Firmicutes ratio as a marker of dysbiosis underscores the need for different and functionally oriented approaches (33). Furthermore, β-diversity – an informative measure for community structure differences – could not be meta-analysed due to inconsistent reporting methods across studies (18, 24–26). Standardised reporting of β-diversity metrics and accessibility of raw sequencing data would greatly enhance comparability in future research. Variability in sequencing region, sequencing depth, and bioinformatic pipelines also contributes to between-study heterogeneity. While subgroup analyses did not reveal major differences between shotgun metagenomics and 16S rRNA sequencing, methodological variation within each approach remains a significant source of inconsistency. From a methodological perspective, restricting the search to English-language full-text articles may have introduced language bias and potentially limited the generalizability of our findings (34), although this approach is consistent with common practice in systematic reviews and meta-analyses (35–37). The secondary computation of the Shannon diversity index from reported taxa and relative abundances may have introduced heterogeneity and potential systematic error. Alpha-diversity estimates are known to be influenced by bioinformatic pipelines, filtering thresholds, taxonomic resolution (ASV/OTU/genus/species), normalization strategies, and sequencing depth, all of which may vary across studies and were not fully harmonizable. Regarding publication bias, the funnel plot for the primary endpoint (α-diversity) is provided in the **Supplementary Material** (**Supplementary File 5**). Visual inspection does not clearly indicate symmetry around the pooled effect size. However, the small number of included studies limits the interpretability of funnel plot asymmetry. Therefore, potential publication bias cannot be excluded. Finally, meta-regression analyses were constrained by the limited number of included studies, which is below the commonly recommended threshold of at least ten studies per covariate. Consequently, these analyses should be considered exploratory and interpreted with caution.

Despite these limitations our study has several strengths. To our knowledge, it provides the first quantitative synthesis examining GM characteristics across both postmenopause and POI. By applying predefined inclusion and exclusion criteria, as well as meta-analytic methods, we aimed to provide a structured and transparent assessment of the available evidence, and to move beyond narrative interpretation, thus establish an objective benchmark for the field. We systematically evaluated heterogeneity, and performed subgroup analyses by sequencing methodology, and by the cause of estrogen deficiency. In addition, we critically considered the limitations of commonly reported microbiome metrics, strengthening the robustness of our conclusions. Finally, our balanced interpretation of null findings and identification of methodological gaps offer a clear roadmap for future research.

Although our results do not support robust GM compositional differences, they do not negate a potential role for the GM in estrogen-related physiology or pathology. Emerging evidence suggests that estrogen-dependent conditions – such as endometriosis – may exhibit disease-specific microbial signatures, and functional pathways may be more relevant than taxonomic composition. Moreover, the role of the microbiome has been also recognized in other aspects of the female reproductive system, including gynecologic malignancies such as endometrial cancer, suggesting its role in the pathogenesis (38).

Our findings highlight the need for future studies that employ standardised analytic pipelines, prioritise GM functional profiling, and focus on well-characterised populations, controlling for known GM disruptors wherever possible.

In conclusion, this systematic review and meta-analysis provide the first quantitative synthesis of GM characteristics in hypoestrogenic versus euestrogenic women. The results do not support consistent differences in α-diversity or broad taxonomic composition associated with estrogen status. However, methodological limitations, substantial heterogeneity, and insufficient control of confounding prevent definitive conclusions. Future high-quality, rigorously controlled studies are needed to better characterise the relationship between estrogen levels and the GM and to define their implications for women’s reproductive, metabolic, and overall health.

## Data Availability

The original contributions presented in the study are included in the article/**Supplementary Material**. Further inquiries can be directed to the corresponding authors.

## Supplementary material

Supplementary File 1 PRISMA Checklist.

Supplementary File 2 Full search strategies for library databases.

Supplementary File 3 List of excluded studies.

Supplementary File 4 Results of quality assessment using the Newcastle-Ottawa Scale.

Supplementary File 5 Funnel Plot for Shannon index.
