# Supplementary figures and images for: The impact of estrogen status on the gut microbiome: a systematic review and meta-analysis
Source: Front Endocrinol (Lausanne). 2026 Apr 2;17:1780806. doi: 10.3389/fendo.2026.1780806 (PMC13082958; doi:10.3389/fendo.2026.1780806)

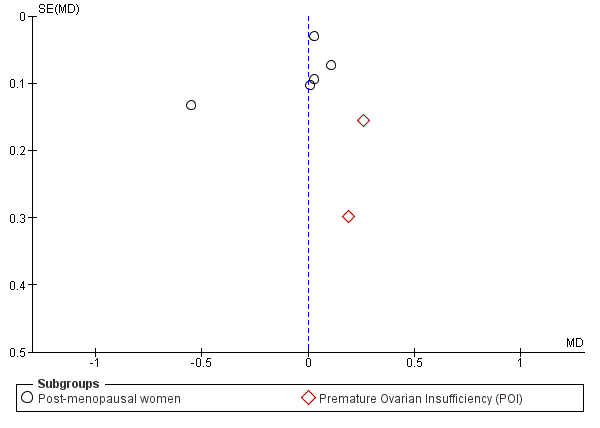

Supplement: Supplementary file 1 [file Image1.png]
