# Supplementary material for: The impact of estrogen status on the gut microbiome: a systematic review and meta-analysis
Source: Front Endocrinol (Lausanne). 2026 Apr 2;17:1780806. doi: 10.3389/fendo.2026.1780806 (PMC13082958; doi:10.3389/fendo.2026.1780806)
Supplement: Supplementary File 1 — PRISMA Checklist. [file SupplementaryFile1.pdf]

FULL SEARCH STRATEGY

PUBMED:
("Premature ovarian insufficiency"[All Fields] OR "POI"[All Fields] OR ("menopause"[MeSH Terms] OR "menopause"[All Fields] OR "menopausal"[All Fields] OR "menopaused"[All Fields] OR "menopauses"[All Fields]) OR ("postmenopausal"[All Fields] OR "postmenopausally"[All Fields] OR "postmenopausals"[All Fields] OR "postmenopause"[MeSH Terms] OR "postmenopause"[All Fields] OR "postmenopausic"[All Fields])) AND ("gut microbiome"[All Fields] OR "gut microbiota"[All Fields] OR "estrobolome"[All Fields])

SCOPUS:
TITLE-ABS-KEY(("Premature ovarian insufficiency" OR POI OR menopause OR postmenopause) AND ("gut microbiome" OR "gut microbiota" OR estrobolome)) AND PUBYEAR < 2025

EMBASE:
('premature ovarian insufficiency' OR poi OR menopause OR postmenopause) AND ('gut microbiome' OR 'gut microbiota' OR estrobolome) AND [01-01-1960]/sd NOT [24-12-2024]/sd AND [<1966-2024]/py
