# Supplementary material for: The impact of estrogen status on the gut microbiome: a systematic review and meta-analysis
Source: Front Endocrinol (Lausanne). 2026 Apr 2;17:1780806. doi: 10.3389/fendo.2026.1780806 (PMC13082958; doi:10.3389/fendo.2026.1780806)
Supplement: Supplementary File 3 — List of excluded studies. [file SupplementaryFile2.pdf]

Supplementary Table 1 - List of excluded studies

**Excluded studies (on full text assessment)**	
**Reference**	**Reason**
Shin et al., 2019 (1)	Hypoestrogenism was not defined
Santos-Marcos et al., 2018 (2)	Missing data on α-diversity
Mayneris-Perxachs et al,. 2020 (3)	Missing data on α-diversity
Flores et al., 2012 (4)	Missing data on α-diversity
Bermingham et al., 2022 (5)	Missing data on α-diversity
Hou et al., 2021 (6)	Missing data on α-diversity
Wang et al., 2022 (7)	Missing data on α-diversity
da Silva et al., 2022 (8)	Missing data on α-diversity
Vriend et al., 2024 (9)	Missing data on α-diversity
Zeber-Lubecka et al., 2024 (10)	Missing data on α-diversity
Li et al., 2024 (11)	Missing data on α-diversity
Annavajhala et al., 2020 (12)	Participants with chronic infection
Feng et al., 2024 (13)	Participants with oncological history
Peters et al., 2021 (14)	Dataset included in a more recent article

References:

1. Shin JH, Park YH, Sim M, Kim SA, Joung H, Shin DM. Serum level of sex steroid hormone is associated with diversity and profiles of human gut microbiome. Res Microbiol. 2019 Jun 1;170(4–5):192–201. doi:10.1016/j.resmic.2019.03.003 PubMed PMID: 30940469.

2. Santos-Marcos JA, Rangel-Zuñiga OA, Jimenez-Lucena R, Quintana-Navarro GM, Garcia-Carpintero S, Malagon MM, et al. Influence of gender and menopausal status on gut microbiota. Maturitas. 2018 Oct 1;116:43–53. doi:10.1016/j.maturitas.2018.07.008 PubMed PMID: 30244778.

3. Mayneris-Perxachs J, Arnoriaga-Rodríguez M, Luque-Córdoba D, Priego-Capote F, Pérez-Brocal V, Moya A, et al. Gut microbiota steroid sexual dimorphism and its impact on gonadal steroids: Influences of obesity and menopausal status. Microbiome. 2020 Sep 20;8(1). doi:10.1186/s40168-020-00913-x PubMed PMID: 32951609.

4. Flores R, Shi J, Fuhrman B, Xu X, Veenstra TD, Gail MH, et al. Fecal microbial determinants of fecal and systemic estrogens and estrogen metabolites: a cross-sectional study [Internet]. 2012. Report. Available from: http://www.translational-medicine.com/content/10/1/253

5. Bermingham KM, Linenberg I, Hall WL, Kad K, Franks PW, Davies R, et al. Menopause is associated with postprandial metabolism, metabolic health and lifestyle: The ZOE PREDICT study. EBioMedicine. 2022;85:104303. doi:10.1016/j

6. Hou MF, Ou-Yang F, Li CL, Chen FM, Chuang CH, Kan JY, et al. Comprehensive profiles and diagnostic value of menopausal-specific gut microbiota in premenopausal breast cancer. Exp Mol Med. 2021 Oct 1;53(10):1636–46. doi:10.1038/s12276-021-00686-9 PubMed PMID: 34707191.

7. Wang Y, Gao X, Lv J, Zeng Y, Li Q, Wang L, et al. Gut Microbiome Signature Are Correlated With Bone Mineral Density Alterations in the Chinese Elders. Front Cell Infect Microbiol. 2022 Mar 31;12. doi:10.3389/fcimb.2022.827575 PubMed PMID: 35433497.

8. da Silva TCA, dos Santos Gonçalves JA, Souza LAC e., Lima AA, Guerra-Sá R. The correlation of the fecal microbiome with the biochemical profile during menopause: a Brazilian cohort study. BMC Womens Health. 2022 Dec 1;22(1). doi:10.1186/s12905-022-02063-8 PubMed PMID: 36474222.

9. Vriend EMC, Galenkamp H, Herrema H, Nieuwdorp M, van den Born BJH, Verhaar BJH. Machine learning analysis of sex and menopausal differences in the gut microbiome in the HELIUS study. NPJ Biofilms Microbiomes. 2024 Dec 1;10(1). doi:10.1038/s41522-024-00628-z

10. Zeber-Lubecka N, Kulecka M, Jagiełło-Gruszfeld A, Dąbrowska M, Kluska A, Piątkowska M, et al. Breast cancer but not the menopausal status is associated with

small changes of the gut microbiota. Front Oncol. 2024;14. doi:10.3389/fonc.2024.1279132

11. Li S, Wang J, Zhang Y, Wang J, Zhou T, Xie Y, et al. Gut microbiota and short-chain fatty acids signatures in postmenopausal osteoporosis patients: A retrospective study. Medicine (United States). 2024 Nov 22;103(47):e40554. doi:10.1097/MD.0000000000040554 PubMed PMID: 39809201.

12. Annavajhala MK, Khan SD, Sullivan SB, Shah J, Pass L, Kister K, et al. Oral and Gut Microbial Diversity and Immune Regulation in Patients with HIV on Antiretroviral Therapy [Internet]. 2020. doi:10.1128/mSphere

13. Feng Y, Zheng H, Yin C, Liang D, Zhang S, Chen J, et al. β-resorcylic acid released by Limosilactobacillus reuteri protects against cisplatin-induced ovarian toxicity and infertility. Cell Rep Med. 2024 Aug;5(8):101678. doi:10.1016/j.xcrm.2024.101678

14. Peters BA, Xue X, Wang Z, Usyk M, Santoro N, Sharma A, et al. Menopausal status and observed differences in the gut microbiome in women with and without HIV infection. Menopause. 2021 May 11;28(5):491–501. doi:10.1097/GME.0000000000001730 PubMed PMID: 33438892.
