# Supplementary material for: The impact of estrogen status on the gut microbiome: a systematic review and meta-analysis
Source: Front Endocrinol (Lausanne). 2026 Apr 2;17:1780806. doi: 10.3389/fendo.2026.1780806 (PMC13082958; doi:10.3389/fendo.2026.1780806)
Supplement: Supplementary File 4 — Results of quality assessment using the Newcastle-Ottawa Scale. [file SupplementaryFile3.pdf]

Supplementary Table 2 - Results of quality assessment using the Newcastle-Ottawa Scale

**Author**	**Zhu et al. 2018** (1)	**Zhao et al. 2019** (2)	**Jiang et al 2021** (3)
Represents cases with independent validation	1	0	1
Cases are consecutive	0	0	0
Controls are from community	0	0	1
Controls have no history of diseases	1	1	1
Controls are comparable for the most important factors	1	1	1
Control for any additional factor	1	1	1
Structured interview where blind to case/control status	1	1	1
Same method of ascertainment for cases and controls	1	1	1
Cases and controls have completed follow up	1	1	1
**Total score**	7	6	8

**Author**	**Wu et al. 2021** (4)	**Yoshikata et al. 2022** (5)	**Peters et al. 2022** (6)	**Wang et al. 2024** (7)
Represents cases with independent validation	1	0	0	0
Cases are consecutive	0	0	0	0
Controls are from community	0	0	1	1
Controls have no history of diseases	1	1	1	1
Controls are comparable for the most important factors	0	0	1	1
Control for any additional factor	0	1	1	1
Structured interview where blind to case/control status	1	1	1	1
Same method of ascertainment for cases and controls	1	1	1	1
Cases and controls have completed follow up	1	1	1	1
**Total score**	5	5	7	7

Newcastle–Ottawa Scale (NOS), which relies on a 9-star system in which scores of 0–3, 4–6, and 7–9 are considered poor, moderate and good quality, respectively

References:

1. Zhu J, Liao M, Yao Z, Liang W, Li Q, Liu J, et al. Breast cancer in postmenopausal women is associated with an altered gut metagenome. Microbiome. 2018 Aug 6;6(1).
2. Zhao H, Chen J, Li X, Sun Q, Qin P, Wang Q. Compositional and functional features of the female premenopausal and postmenopausal gut microbiota. FEBS Lett. 2019 Sep 1;593(18):2655–64.
3. Jiang L, Fei H, Tong J, Zhou J, Zhu J, Jin X, et al. Hormone Replacement Therapy Reverses Gut Microbiome and Serum Metabolome Alterations in Premature Ovarian Insufficiency. Front Endocrinol (Lausanne). 2021 Dec 23;12.
4. Wu J, Zhuo Y, Liu Y, Chen Y, Ning Y, Yao J. Association between premature ovarian insufficiency and gut microbiota. BMC Pregnancy Childbirth. 2021 Dec 1;21(1).
5. Yoshikata R, Yamaguchi M, Mase Y, Tatuzuki A, Myint KZ, Ohta H. Age-related changes, influencing factors and crosstalk between vaginal and gut microbiota: a cross-sectional comparative study of pre- and postmenopausal women [Internet]. 2021. Available from: http://medrxiv.org/lookup/doi/10.1101/2021.12.22.21268221
6. Peters BA, Lin J, Qi Q, Usyk M, Isasi CR, Mossavar-Rahmani Y, et al. Menopause Is Associated with an Altered Gut Microbiome and Estrobolome, with Implications for Adverse Cardiometabolic Risk in the Hispanic Community Health Study/Study of Latinos. mSystems. 2022 Jun 28;7(3).
7. Wang Y, Sharma A, Weber KM, Topper E, Appleton AA, Gustafson D, et al. The menopause-related gut microbiome: Associations with metabolomics, inflammatory protein markers, and cardiometabolic health in women with HIV. Menopause. 2024 Jan 1;31(1):52–64.
